# TElmisartan in the management of abDominal aortic aneurYsm (TEDY): The study protocol for a randomized controlled trial

**DOI:** 10.1186/s13063-015-0793-z

**Published:** 2015-06-17

**Authors:** Dylan R. Morris, Margaret A. Cunningham, Anna A. Ahimastos, Bronwyn A. Kingwell, Elise Pappas, Michael Bourke, Christopher M. Reid, Theo Stijnen, Ronald L. Dalman, Oliver O. Aalami, Jan H. Lindeman, Paul E. Norman, Philip J. Walker, Robert Fitridge, Bernie Bourke, Anthony E. Dear, Jenna Pinchbeck, Rene Jaeggi, Jonathan Golledge

**Affiliations:** Queensland Research Centre for Peripheral Vascular Disease, School of Medicine and Dentistry, James Cook University, Townsville, QLD Australia; Psychology Department, University of Stirling, Stirling, UK; Baker IDI Heart and Diabetes Institute and The Department of Cardiovascular Medicine, Alfred Hospital Melbourne, Melbourne, Australia; Gosford Vascular Services, Gosford, New South Wales Australia; Centre of Cardiovascular Research and Education in Therapeutics, Department of Epidemiology and Preventive Medicine, Monash University, Alfred Hospital, Melbourne, Australia; Leiden University Medical Center, Leiden, The Netherlands; Division of Vascular Surgery, Department of Surgery, Stanford University School of Medicine, Stanford, CA USA; School of Surgery, University of Western Australia, Perth, WA Australia; University of Queensland School of Medicine, Discipline of Surgery and Centre for Clinical Research, and Department of Vascular Surgery, Royal Brisbane and Women’s Hospital, Brisbane, Queensland Australia; Department of Surgery, University of Adelaide, The Queen Elizabeth Hospital, Adelaide, South Australia Australia; Eastern Health Clinical School, Department of Medicine, Monash University, Melbourne, Australia; The Department of Vascular and Endovascular Surgery, The Townsville Hospital, Townsville, QLD Australia

**Keywords:** Abdominal aortic aneurysm, Trial, Angiotensin, Telmisartan

## Abstract

**Background:**

Experimental studies suggest that angiotensin II plays a central role in the pathogenesis of abdominal aortic aneurysm. This trial aims to evaluate the efficacy of the angiotensin receptor blocker telmisartan in limiting the progression of abdominal aortic aneurysm.

**Methods/Design:**

Telmisartan in the management of abdominal aortic aneurysm (TEDY) is a multicentre, parallel-design, randomised, double-blind, placebo-controlled trial with an intention-to-treat analysis. We aim to randomly assign 300 participants with small abdominal aortic aneurysm to either 40 mg of telmisartan or identical placebo and follow patients over 2 years. The primary endpoint will be abdominal aortic aneurysm growth as measured by 1) maximum infra-renal aortic volume on computed tomographic angiography, 2) maximum orthogonal diameter on computed tomographic angiography, and 3) maximum diameter on ultrasound. Secondary endpoints include change in resting brachial blood pressure, abdominal aortic aneurysm biomarker profile and health-related quality of life. TEDY is an international collaboration conducted from major vascular centres in Australia, the United States and the Netherlands.

**Discussion:**

Currently, no medication has been convincingly demonstrated to limit abdominal aortic aneurysm progression. TEDY will examine the potential of a promising treatment strategy for patients with small abdominal aortic aneurysms.

**Trial registration:**

Australian and Leiden study centres: Australian New Zealand Clinical Trials Registry ACTRN12611000931976, registered on 30 August 2011; Stanford study centre: clinicaltrials.gov NCT01683084, registered on 5 September 2012.

## Background

Abdominal aortic aneurysm (AAA) is a major contributor to mortality in western countries, responsible for over 10,000 annual deaths in the USA and costing the healthcare system more than $3 billion per annum [1, 2]. Over the last decade a number of advances have been made to the management of AAA including the introduction of screening programs designed to identify AAAs before they rupture and the development of sophisticated minimally invasive techniques to repair large AAAs [3, 4]. The introduction of targeted AAA ultrasound screening programs in a number of countries, such as the United States, the United Kingdom and Sweden, is expected to reduce AAA-related mortality. Individuals found to have large AAAs (infra-renal aortic diameter >55 mm) are usually considered for elective surgical repair to prevent potentially fatal AAA rupture. However 90 % of AAAs detected through screening are <55 mm in diameter (small AAAs), and these carry a low risk of rupture [5, 6]. Four trials have suggested that early elective open or endovascular repair does not reduce mortality for patients with AAAs measuring 40 to 55 mm in diameter [7]. Current guidelines therefore advocate that patients with small AAAs be managed conservatively with surveillance comprising repeated imaging and consultations [8]. On average, small AAAs expand 1 to 3 mm per year and up to 70 % of patients will eventually require surgery [9–11]. This current practice of simply monitoring small AAAs is associated with reduced health-related quality of life, possibly due to patients’ concerns that no active treatment is being undertaken [12].

Effective drug therapies that prevent or limit AAA growth may improve health-related quality of life, reduce the need for surgery and prevent mortality in patients unsuitable for AAA repair. There is limited evidence suggesting that the control of atherogenic risk factors alone slows AAA progression [13]. However such treatment is still advised as these individuals are at high risk of acute cardiovascular events [14]. A number of small randomised controlled trials have been conducted in humans in an attempt to discover a drug that limits AAA growth [15, 16]. Only two published studies, using the β-blocker propranolol and the antibiotic doxycycline, included more than 200 patients, and neither study reported a benefit of the medication tested [17, 18]. Drugs shown to slow AAA progression in animal models and observational human studies include statins, macrolides, cyclooxygenase inhibitors and developmental agents such as c-Jun N-terminal kinase inhibitors [19–23]. While statins are well tolerated, they are already indicated and prescribed in most patients with AAAs and therefore difficult to assess for their efficacy in limiting AAA progression [24, 25]. A potential drug to be trialled for AAA ideally requires a number of important characteristics including evidence to support its benefit, a good safety profile from phase II studies or use of the medication in related conditions, and likelihood of good compliance.

There is a large body of evidence suggesting that angiotensin II plays a critical role in AAA formation, progression and rupture [26]. Infusion of angiotensin II via a subcutaneous osmotic pump constitutes a commonly applied mouse model of aortic aneurysm, utilized in hundreds of studies to date [27]. Such angiotensin II-induced AAAs have some features comparable to human AAA, including a similar gene expression profile on microarray analysis; marked inflammatory focus; associated thrombus; and predilection for males and sites of atherosclerosis [28]. Angiotensin II activates the angiotensin II receptor type 1 (AT1) to promote a series of molecular changes implicated in AAA formation including up-regulation of pro-inflammatory cytokines and proteins, such as osteopontin (OPN), osteoprotegerin (OPG), transforming growth factor-beta1 (TGF-β1), and matrix metalloproteinase-9 (MMP-9) [29–31]. The reduction in ascending aortic diameter in patients with Marfan syndrome after 6 months therapy with the angiotensin converting enzyme inhibitor, perindopril, supports a clinical rationale for renin angiotensin system inhibition in humans [32]. The 3 to 7 mm reduction in aortic diameter in this trial was also associated with reduction in circulating TGF-β and MMP levels [33].

Given the current evidence suggesting a central role of angiotensin II in the pathogenesis of AAA, the use of AT1 blockers to potentially slow AAA progression has generated considerable interest. Telmisartan is a potent, long-acting AT1 blocker classically indicated for essential hypertension, heart failure and renal failure. We hypothesise that telmisartan will reduce AAA growth by down-regulation of the AT1 pathway. Telmisartan also has PPARγ agonist activity that is greater than other AT1 blockers [34, 35]. Evidence has accumulated showing PPARγ ligation to inhibit angiotensin II-mediated up-regulation of OPN, OPG, MMP-9 and TGF-β1, which are implicated in AAA [36–39]. In animal models PPARγ agonists limit AAA development, progression and rupture [36, 38, 40]. The combination of AT1 blockade and PPARγ ligation could be very effective in limiting AAA expansion in humans.

Inhibition of AT1 could have benefit over angiotensin-converting enzyme (ACE) inhibition for several reasons: (i) ACE inhibition, unlike AT1 blockade, only indirectly inhibits the angiotensin pathway; (ii) a number of non-ACE angiotensin II producing enzymes have been identified in AAA biopsies, suggesting that ACE inhibition may only partially inhibit the effects of angiotensin II [41, 42]; (iii) part of the blood pressure-lowering effect of ACE inhibitors is attributable to an increase in circulating kinins, which have unknown effects on AAA [43]; and (iv) AT1 blockers demonstrate fewer and less severe side effects compared to ACE inhibitors in some studies [44].

Telmisartan has been shown to inhibit AAA in three different animal model studies [45–47]. AT1 blocker prescription has also been associated with reduced AAA progression in one surveillance study involving 1,269 patients with small AAA [48]. To date, no randomised trials have been conducted to examine the effect of an AT1 inhibitor on AAA progression. This trial seeks to evaluate the efficacy of telmisartan in limiting AAA expansion.

## Methods/Design

### Study design

Telmisartan in the management of abdominal aortic aneurysm (TEDY) is a parallel-design, randomised, double-blind, placebo-controlled trial to assess whether treatment with telmisartan for 2 years will reduce the rate of AAA expansion. The TEDY trial plans to enrol 300 participants with small AAA, defined by a maximum orthogonal infra-renal aortic diameter of ≥35 and ≤49 mm on computed tomographic angiography (CTA) or ultrasound. In order to explicitly assess the impact of AT1 blockade on AAA expansion, TEDY will only include individuals who have no current or planned usage of AT1 blockers or ACE inhibitors. TEDY will not include individuals with an existing indication for AAA repair (according to the treating physician) or expectation that this will be revised within one year. Furthermore, participants will only be included if they have a high likelihood of treatment compliance for 24 months according to their supervising physician. Additional exclusion criteria include contraindications to telmisartan and previous abdominal aortic surgery. A full list of inclusion and exclusion criteria is given in Table 1.

**Table 1:** Patient eligibility criteria

**Inclusion criteria**
• Written informed consent
• Infra-renal AAA with maximum orthogonal diameter of ≥35 mm and ≤49 mm on CTA or ultrasound
• No current indication for AAA repair, or expectation that this will be revised in 12 months
• Likely to comply with treatment over 24 months, including seven visits to study centre
**Exclusion criteria**
• Currently taking or likely to start taking an AT1 blocker
• Currently taking or likely to start taking an ACE inhibitor
• Previous abdominal aortic surgery
• Contraindication to telmisartan:
o Renal impairment: creatinine >1.5 x ULN
o Known renal artery stenosis >70 % of one or both renal arteries
o Chronic liver disease (that is, cirrhosis or hepatitis) or abnormal liver function (that is, ALT > 1.5 x ULN)
o Electrolyte imbalance
o Active gout

*AAA,* abdominal aortic aneurysm; ACE, angiotensin converting enzyme; ALT, alanine transaminase; *AT1,* angiotensin II receptor type 1; CTA, computed tomographic angiography; *ULN,* upper limit of normal

### Randomisation and follow-up

**Fig. 1:**
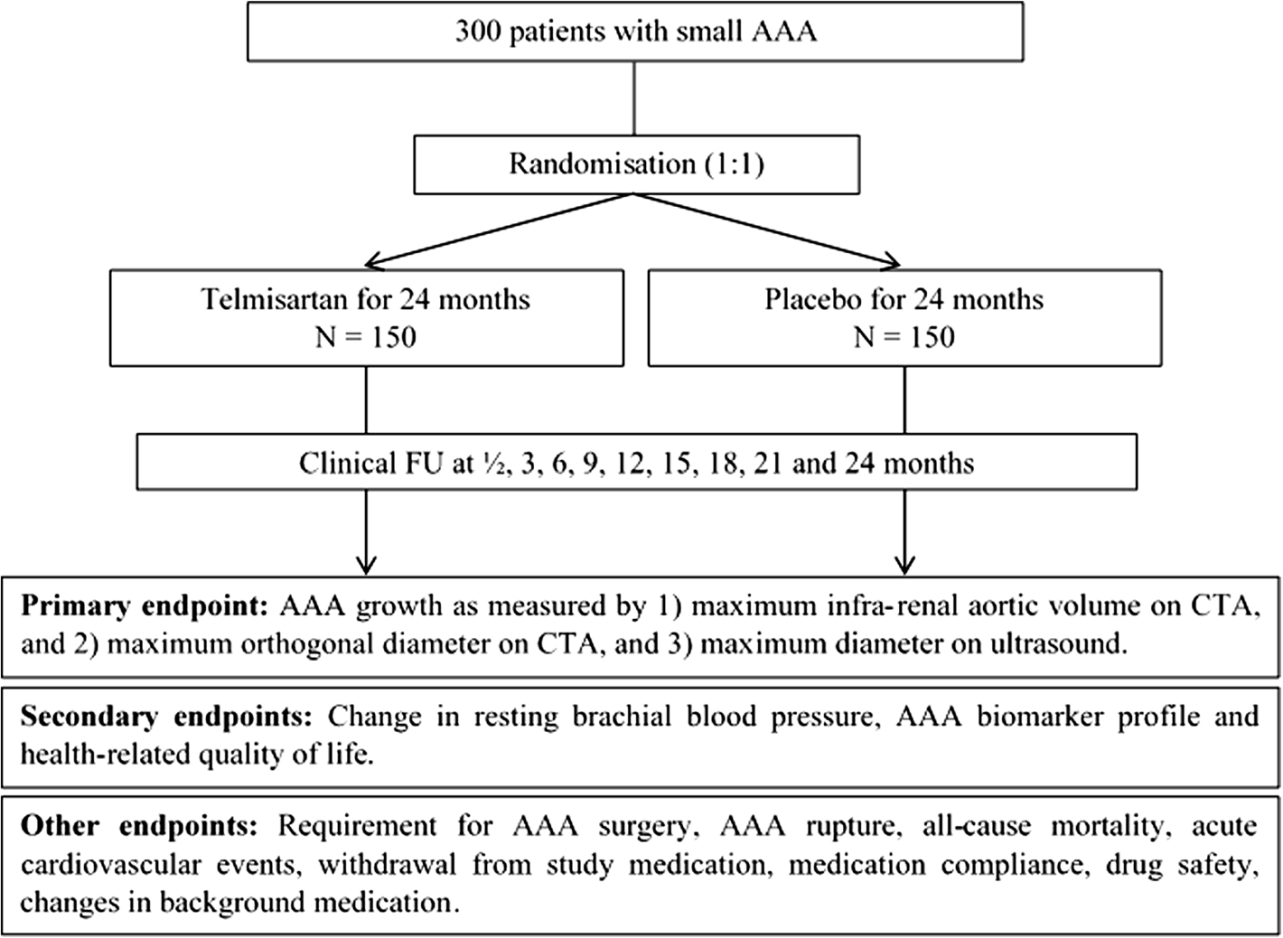
Design of the Telmisartan in the management of abdominal aortic aneurysm (TEDY) Trial

The overall design of the TEDY trial is shown in Fig. 1. At the initial visit, individuals will be considered for inclusion in the trial (Table 1) and if eligible, informed consent will be sought. Individuals will undergo a short health survey, clinical examination by a medical officer, resting brachial blood pressure, resting ankle-brachial index, and collection of fasting blood samples for analysis of lipid profile, blood glucose, inflammatory markers, AAA biomarkers, liver function and renal function. An additional blood sample will be stored for future genetic, protein and lipidomic analyses. A baseline CTA and ultrasound will be obtained for detailed measurements of the AAA.

Eligible participants who have provided written informed consent will return for a randomisation visit, during which they will be allocated to either telmisartan (40 mg od daily) or matching placebo (once daily) for 24 months. Patients will be randomised by a blinded health professional through the secure trial website, coordinated by an independent trial centre. Treatment allocation will be determined by a computer-generated random number sequence that is stratified by study site and initial aortic diameter (35 to 39 mm, 40 to 44 mm, and 45 to 49 mm). Random number sequences will correspond to serial numbers on concealed study packaging. Only the trial drug centre and the Data and Safety Monitoring Board (DSMB) will know the identity of pre-packaged drugs. The Stanford study centre will utilize an independent randomization procedure implemented locally, whereby ID numbers are randomly allocated to the two study groups using randomization software (www.tufts.edu/~gdallal/randomize.htm). The resultant randomization key will be provided to the on-site pharmacy, which dispenses study medication to participants according to the condition assignment prescribed by the key.

Following randomisation, participants will receive their allocated study medication and subsequent instruction on the dosing regimen. Individuals will be contacted by phone 2 weeks after randomisation and asked about adherence to study treatment and known medication side effects. Phone consultations will be repeated at 9, 15 and 21 months. Follow-up visits will occur at 3, 6, 12, 18 and 24 months, during which the occurrence of trial endpoints and adverse events will be appraised. Participants will receive ongoing health questionnaires, clinical examination, blood tests and imaging according to Table 2. All participants, including those in whom allocated medication is discontinued, will be invited to follow up for the duration of the study in the absence of AAA repair. Final follow-up will occur at 24 months after randomisation, at which point the participant will discontinue the prescribed medication.

**Table 2:** Visit schedule

	Study time points (months)										
	0	0	½	3	6	9	12	15	18	21	24
Assessment	Visit 1	Visit 2	Phone Call	Visit 3	Visit 4	Phone Call	Visit 5	Phone Call	Visit 6	Phone Call	Visit 7
Consent	x										
Enrolment/randomisation		x									
CT angiography	x						x^a^				x
Ultrasound	x				x		x		x		x
Blood test (safety)	x			x			x				x
Blood collection (study)	x				x		x		x		x
Resting brachial BP	x			x	x		x		x		x
SF-36 questionnaire	x						x				x
Physical assessments	x^±^			x	x		x		x		x
Medical examination	x			x			x				x
Resting ABI	x						x				x
Collection of study medication		x			x		x		x		
Return of study medication					x		x		x		x
Event outcomes				x	x		x		x		x
AE and compliance check			x	x	x	x	x	x	x	x	x

Physical assessments include: resting BP, resting heart rate, weight, hip and waist measurements, ^±^indicates an additional assessment of height. ABI, ankle brachial-index; AE, adverse effects; BP, blood pressure; CT, computed tomography

^a^The Leiden and Stanford study centres will not perform 12-month CTAs due to local ethics regulations. The Stanford centre will utilize a slightly different study schedule. In place of the 2-week phone call and 3-month visit, participants will attend for a safety visit at 1 month for assessment of adherence to study treatment and medication side effects. Study medications will be prescribed at 3-month intervals with AE and compliance checks at 3, 9, 15 and 21 months

### Endpoints

The trial primary endpoint will be the difference in AAA growth between the intervention and control groups over 24 months. AAA growth will be assessed by 1) maximum infra-renal aortic volume on CTA, 2) maximum orthogonal diameter on CTA, and 3) maximum diameter on ultrasound. Infra-renal aortic volume can be measured in a reproducible way and changes in it appear to provide a more sensitive means to assess AAA growth [49]. It is expected that a number of participants may not receive a CTA at 24 months due to drop-out, for example due to requirement for AAA surgery. We therefore have also included ultrasound measured AAA growth as a primary endpoint since ultrasound assessments will be performed every 6 months.

CTA will be obtained at entry, 12 and 24 months depending on local ethics guidelines (CTA at entry and 24 months only for Stanford and Leiden study centres). Assessment of infra-renal aortic volume will be performed according to a previously established protocol at the main study centre (mean inter-observer coefficient of variation = 2.7 %) [49]. The total volume of the infra-renal aorta, including the aortic wall and lumen, will be estimated using a semi-automated program that we have previously established to be reproducible [49]. In short, axial regions of interest are drawn around the aorta from the slice inferior to the origin of the lowest renal artery to the slice superior to the bifurcation of the aorta. The areas of these regions of interest are summated using CT analysis software to yield an overall volume. The maximum orthogonal AAA diameter on CTA is a measurement of infra-renal aortic diameter perpendicular to the lumen, devised to avoid over-estimation of tortuous AAAs. We have previously reported a reproducible protocol to measure orthogonal aortic diameter (mean inter-observer coefficient of variation = 3.5 %) [50]. Initially, seeds are placed in the centre of the infra-renal aorta from the renal arteries to the aortic bifurcation. Curved multi-planar reconstruction is then used to reconstruct the aorta perpendicular to its axis. The reconstructed aorta is subsequently scouted to find the region of maximum diameter, taking many measurements. The largest diameter is taken to be the maximum orthogonal diameter.

Ultrasound measurements will be undertaken at entry, 6, 12, 18 and 24 months. Maximum anteroposterior and transverse infra-renal aortic diameter will be measured by experienced sonographers using a 3.75-MHz transducer and ultrasound machines present in vascular laboratories of each centre. Diameters will be measured in the plane of the aorta with callipers placed on the outer part of the aortic wall. The largest external anterior-posterior and transverse AAA diameters will be recorded. The location at which the maximum diameter is measured will always be within the infra-renal aorta but will be selected based on the maximum diameter not particular location within this artery segment. The intra-observer reproducibility of ultrasound measurements has been assessed at major participating centres and shown to be good (coefficient of repeatability ≤1.6 mm for four reporting centres) [18, 31]. Central analysis of both CTA and ultrasound images will be performed at the study centre.

Secondary endpoints include change in resting brachial blood pressure, AAA biomarker profile, and health-related quality of life. Resting brachial blood pressure will be measured at entry, 3, 6, 12, 18 and 24 months. Three blood pressure measurements will be recorded at 3-minute intervals, with the average blood pressure used as the representative value. Blood will be taken at entry, 6, 12, 18 and 24 months to assess changes in circulating AAA biomarkers. The AAA biomarker panel will include OPN, OPG, D-dimer, TGF-β1 and MMP-9, and will be measured centrally. These circulating proteins have been selected based on extensive evidence suggesting that they have a central role in AAA pathology [39, 51]. Health-related quality of life will be assessed by the Short Form-36 (SF-36) questionnaire collected at entry, 12 and 24 months. The SF-36 has previously been validated for use in elderly patients with vascular disease [52, 53].

Additional outcome data recorded will include requirement for AAA repair surgery as determined by the treating vascular surgeon, AAA rupture, all-cause mortality, acute cardiovascular events, withdrawal from study medication, drug safety and changes in background medication. Medication compliance will be assessed by hand-counting of pills during scheduled visits.

### Sample size and power calculations

At present there is insufficient preliminary data demonstrating an accurate effect size of AT1 blockers on AAA growth. In line with other AAA drug trials, we estimated our sample size based on a clinically relevant reduction of 30 % in annual AAA growth [18]. Given local AAA growth rates (mean = 1.20 mm/y, standard deviation = 1.16) applicable to the study population, we calculated an effect size d = 0.36 [54]. Accordingly, based on t-test analysis, this study requires 126 participants per group (252 in total, power = 80 %, α = 0.05) to detect the hypothesised reductions in AAA growth. Taking into account a 24-month drop-out rate of approximately 20 %, we plan to recruit 300 patients.

### Study organization

The TEDY trial has been developed through an international collaboration of experts in AAA biology, clinical management of AAA, aortic imaging and drug trials. TEDY is a multi-centre study conducted from major vascular centres in Australia, The Netherlands and the USA. The study steering committee comprises senior vascular investigators from the centres involved and experts in drug trials and data analysis (Appendix). This will be the main policy and decision-making committee for the study and will meet by teleconference. Currently, there are nine study centres in total, seven in Australia, one in Leiden, Netherlands, and one in Stanford, California, USA. The Stanford study centre is sponsored by Medtronic Inc. (Santa Rosa, CA) and will utilise independent randomisation, medication concealment and imaging analysis. The core imaging and biomarker laboratories are located at the Vascular Biology Unit, Queensland Research Centre for Peripheral Vascular Disease, James Cook University, Townsville Australia, and the Veteran’s Administration Palo Alto Health Care System, USA. The web-based randomisation database will be managed by the Monash Clinical Trials Centre, Melbourne Australia, with an independent randomisation centre at Stanford University. The Baker IDI Heart and Diabetes Institute will coordinate medication concealment and packaging for Australian centres. The Leiden study centre will coordinate medication concealment locally for convenience. An independent DSMB will review all adverse events and conduct an interim analysis.

### Data handling

Trial documentation including design, eligibility criteria, protocols and case report forms (CRF) will be shared electronically with participating study centres. The online clinical trial portal will house the central web-based randomisation system (https://ccre.med.monash.edu.au/TedyRand/). Data recorded on printed CRFs will be faxed or scanned to the trial coordinating centre where it will be entered centrally and examined for data quality. All participating centres have received local ethics approval and the study is being conducted in accordance with the Declaration of Helsinki. The TEDY trial is registered at www.anzctr.org.au: ACTRN12611000931976. The Stanford arm of the trial is registered at www.clinicaltrials.gov ID: NCT01683084.

### Safety

Previous large randomised control trials have shown telmisartan to have a good safety record [55, 56]. The TRANSCEND trial demonstrated negligible side effects for telmisartan (80 mg od daily) in approximately 6,000 individuals with cardiovascular disease [55]. These findings suggest that telmisartan (40 mg od daily) should be well tolerated by the majority of patients eligible for the TEDY trial. Participant safety will be assessed on all visits and phone calls. Detailed safety assessment will occur at 3, 12 and 24 months and will involve a targeted medical history, physical examination of heart rate and brachial blood pressure, laboratory parameters including serum chemistry (liver function tests, glucose, creatinine, sodium, potassium, chloride) and haematology (haemoglobin, differential white blood cell count and platelet count), and adverse event reporting via report forms provided to study centres. Participant safety will also be assessed by phone calls occurring at 2 weeks, 9 months, 15 months and 21 months. Adverse events may comprise shortness of breath, wheeze, rash, dizziness and fatigue. Possible side effects of the medication include back pain, diarrhoea, respiratory tract infection and sinus inflammation. In line with other medication trials, serious adverse events (SAEs) will be defined as death, requirement for in-patient hospital treatment and persistent or significant disability.

The DSMB will consist of an independent epidemiologist, vascular surgeon and statistician; and will meet to assess all adverse events. In particular, the DSMB will concentrate on AAA-related events such as sudden death due to AAA rupture, as well as adverse events related to study medication. Expected AAA rupture rates for small AAAs are approximately 1 to 2 % per year based on published data [9, 10]. Rupture rates greater than this will be considered to be a cause for concern. In this instance consideration will be given to assessment of all other outcome data from the study. AAA growth will be compared between placebo and telmisartan groups using this data. The final decision to stop the study will take into account AAA ruptures, AAA growth data and any other serious complications. Following their assessment, the DSMB would report their recommendation to the steering committee. One formal interim analysis will be undertaken when 60 % of participants have been followed-up for 24 months. Based on all available data at the time of the interim analysis, the difference in growth at 24 months of follow-up will be estimated and tested.

### Statistical analysis

The principal analysis of primary and secondary endpoints will be based on intention to treat at the time of randomisation. All patients who meet the eligibility criteria, provide written informed consent and are enrolled in the study will be included in the primary analysis, regardless of adherence to treatment medication. The efficiency of randomisation will be assessed by comparing the distribution of recognized determinants of AAA progression and also background medication use prior to the study between the treatment and control groups.

The primary efficacy parameter is the between treatment groups difference in mean growth in AAA over 2 years as estimated by the maximum AAA volume on CTA, maximum orthogonal diameter on CTA, and maximum diameter on ultrasound. If there were to be no drop-outs, the observed mean progression over 2 years would be compared and tested using a two-sample t-test. However, it is expected that a non-negligible proportion of patients will drop out before 2 years due to aneurysm repair. Aneurysm growth will therefore be analysed by a linear mixed model, specifying zero difference between treatment groups at baseline. The model will have up to 11 repeated dependent measurements: three CTA volume measurements, three CTA diameter measurements, and five ultrasound measurements.

The fixed part of the model will contain the following covariates: Time for CTA measurements as a categorical, time for ultrasound measurements as a categorical, treatment, interaction between treatment and CTA time, and interaction between treatment and ultrasound time. The covariance part of the model will constitute a linear covariance matrix containing the following parameters: Variance of CTA measurements, variance of ultrasound measurements and covariance parameters. Without reference to the fixed part of the model, the goodness of fit of the assumed covariance model will be investigated. If necessary, the model will be extended with additional terms. The analysis will be performed using the PROC MIXED procedure on SAS (Cary, North Carolina), which is capable of fitting arbitrary linear covariance matrices. The main null hypothesis that will be tested is based on the treatment effect as estimated by this model at 24 months on CTA and ultrasound measurements.

It is possible that participants randomised to telmisartan may have a small reduction in blood pressure. In the event that the control group are more frequently receiving other blood pressure modifying medications at the completion of the trial, post-hoc analyses will be conducted with appropriate adjustment for these confounding factors.

The stopping boundaries are based on the alpha and beta spending function theory and the stopping rule preserves the overall probability of 0.05 of rejecting the null hypothesis in favour of telmisartan while the null hypothesis is true [57]. The stopping rule is flexible in the sense that the actual time of the interim analyses may deviate from the scheduled time and that more interim analyses may be inserted. The stopping rule is designed using SAS PROC SEQDESIGN. We have chosen a gamma cumulative spending function with gamma parameter equal to −5 for alpha and −7 for beta. These choices lead to conservative stopping boundaries, such that the trial will stop for superiority only if the evidence for a favourable telmisartan effect is very strong and the trial will stop for futility only if the interim analysis suggests it very unlikely that a significant telmisartan effect will be found at the end of the trial. At the interim analysis, the trial will stop for superiority if the one-sided *P* value in favour of telmisartan is smaller than 0.0032. The trial will stop for futility if the one-sided *P* value is larger than 0.5.

## Discussion

A number of randomised control trials have been undertaken to examine the efficacy of AAA growth-limiting drugs. To date, no medication has demonstrated efficacy in reducing AAA growth convincingly. TEDY is the first clinical trial to address whether AT1 blockers can limit AAA progression in humans. Another trial is assessing the influence of an ACE inhibitor on AAA progression [58]; however, the mode of action of AT1 blockers and ACE inhibitors are not analogous. A large population study reported that ACE inhibitor prescription was associated with less likelihood of presenting with a ruptured AAA [59]. Another study, however, found ACE inhibitor prescription to be associated with increased AAA expansion [60]. There is preliminary evidence from animal studies, observational studies in humans, and randomised trials on Marfan’s disease to suggest that AT1 blockade will limit AAA growth in humans [32, 45–48]. If successful, TEDY will confirm the central role of the angiotensin pathway in AAA pathogenesis, and provide the first growth limiting drug for secondary prevention of AAA.

## Trial status

Participants are currently being recruited.

## Appendix

**Current study centres:** (1) The Alfred Hospital, Melbourne: Dr Anna Ahimastos, Professor Bronwyn Kingwell, and Dr Wayne Childs; (2) Fremantle Hospital, Perth: Professor Paul Norman; (3) Gosford Vascular Services, Gosford: Dr Bernie Bourke and Mr Michael Bourke; (4) Queen Elizabeth Hospital, Adelaide: Professor Robert Fitridge, Dr Prue Cowled, and Ms Ruth Battersby; (5) Royal Brisbane and Women’s Hospital, Brisbane: Professor Philip Walker, Dr Jason Jenkins, Dr Sophie Rowbotham, Dr Brad Stefanovic, and Ms Rachael Nowak; (6) The Mater Hospital, Townsville: Dr Francis Quigley and Ms Barbara Bradshaw; (7) The Townsville Hospital, Townsville: Professor Jonathan Golledge, Dr Rene Jaeggi, Mrs Jenna Pinchbeck, Ms Leonie Jones, Mrs Elise Pappas, and Ms Barbara Bradshaw; (8) Leiden University Medical Center, Leiden, The Netherlands: Associate Professor Jan Lindeman and Stephanie Tomee; (9) Veterans Administration Hospital & Stanford University, Palo Alto, CA, USA: Dr Ronald L. Dalman, Dr Oliver O. Aalami, and Ms Lori K. McDonnell.

**Trial Randomisation Centres:** Monash Clinical Trials Centre, Melbourne Australia; and Stanford University.

**Drug Coordinating Centres:** Baker IDI, Melbourne Australia; and Leiter’s Pharmacy and Veteran’s Administration Palo Alto Health Care System Pharmacy, CA, USA.

**Core Imaging and Biomarker Laboratories:** Department of Vascular and Endovascular Surgery, The Townsville Hospital and James Cook University, Townsville, Australia; and Veteran’s Administration Palo Alto Health Care System, CA, USA.

**Steering Committee:** Dr Anna Ahimastos, Dr Ronald L. Dalman, Professor Jonathan Golledge, Professor Bronwyn Kingwell, Associate Professor Jan Lindeman, Professor Paul Norman, Professor Christopher Reid, and Professor Philip Walker.

**Data and Safety Monitoring Board:** Professor Andrew Tonkin, Dr Jack Walsh, and Professor Theo Stijnen.

**Publications:** Dr Anna Ahimastos, Professor Jonathan Golledge, Professor Bronwyn Kingwell, Professor Paul Norman, Professor Philip Walker, Dr Jan Lindeman, and Dr Ronald Dalman.

## Abbreviations

AAA: abdominal aortic aneurysm
ACE: angiotensin-converting enzyme
AT1: angiotensin II receptor type 1
CRF: case report form
CTA: computed tomographic angiography
DSMB: Data and Safety Monitoring Board
MMP-9: matrix metalloproteinase-9
OPG: osteoprotegerin
OPN: osteopontin
SAE: serious adverse event
SF-36: Short Form-36
TEDY: Telmisartan in the management of abdominal aortic aneurysm
TGF-β1: transforming growth factor-beta1

## References

[1] Hoyert DL, Xu JQ (2012). Deaths: preliminary data for 2011. National vital statistics reports; vol 61 no 6.

[2] Agency for Healthcare Research and Quality (2006). Hospital cost and utilization project: nationwide inpatient sample data set.

[3] Buxton MJ (2009). Screening for abdominal aortic aneurysm. BMJ.

[4] United Kingdom ETI, Greenhalgh RM, Brown LC, Powell JT, Thompson SG, Epstein D (2010). Endovascular versus open repair of abdominal aortic aneurysm. N Engl J Med.

[5] Ashton HA, Buxton MJ, Day NE, Kim LG, Marteau TM, Scott RA (2002). The multicentre aneurysm screening study (MASS) into the effect of abdominal aortic aneurysm screening on mortality in men: a randomised controlled trial. Lancet.

[6] Norman PE, Jamrozik K, Lawrence-Brown MM, Le MT, Spencer CA, Tuohy RJ (2004). Population based randomised controlled trial on impact of screening on mortality from abdominal aortic aneurysm. BMJ.

[7] Filardo G, Powell JT, Martinez MA, Ballard DJ (2012). Surgery for small asymptomatic abdominal aortic aneurysms. Cochrane Database Syst Rev.

[8] Rooke TW, Hirsch AT, Misra S, Sidawy AN, Beckman JA, Findeiss LK (2011). 2011 ACCF/AHA focused update of the guideline for the management of patients with peripheral artery disease (updating the 2005 guideline): a report of the american college of cardiology foundation/american heart association task force on practice guidelines. J Am Coll Cardiol.

[9] Lederle FA, Wilson SE, Johnson GR, Reinke DB, Littooy FN, Acher CW (2002). Immediate repair compared with surveillance of small abdominal aortic aneurysms. N Engl J Med.

[10] United Kingdom Small Aneurysm Trial P (2002). Long-term outcomes of immediate repair compared with surveillance of small abdominal aortic aneurysms. N Engl J Med.

[11] Powell JT, Sweeting MJ, Brown LC, Gotensparre SM, Fowkes FG, Thompson SG (2011). Systematic review and meta-analysis of growth rates of small abdominal aortic aneurysms. Br J Surg.

[12] Small UK (1998). Aneurysm trial participants. Health service costs and quality of life for early elective surgery or ultrasonographic surveillance for small abdominal aortic aneurysms. Lancet.

[13] Brady AR, Thompson SG, Fowkes FG, Greenhalgh RM, Powell JT (2004). UK small aneurysm trial participants. Abdominal aortic aneurysm expansion: risk factors and time intervals for surveillance. Circulation.

[14] Powell JT, Brown LC, Forbes JF, Fowkes FG, Greenhalgh RM, Ruckley CV (2007). Final 12-year follow-up of surgery versus surveillance in the UK Small Aneurysm Trial. Br J Surg.

[15] Rughani G, Robertson L, Clarke M (2012). Medical treatment for small abdominal aortic aneurysms. Cochrane Database Syst Rev.

[16] Golledge J, Norman PE (2011). Current status of medical management for abdominal aortic aneurysm. Atherosclerosis.

[17] Propanolol Aneurysm Trial I (2002). Propranolol for small abdominal aortic aneurysms: results of a randomized trial. J Vasc Surg.

[18] Meijer CA, Stijnen T, Wasser MN, Hamming JF, van Bockel JH, Lindeman JH (2013). Doxycycline for stabilization of abdominal aortic aneurysms: a randomized trial. Ann Intern Med.

[19] Golledge J, Muller J, Daugherty A, Norman P (2006). Abdominal aortic aneurysm: pathogenesis and implications for management. Arterioscler Thromb Vasc Biol.

[20] Schouten O, van Laanen JH, Boersma E, Vidakovic R, Feringa HH, Dunkelgrun M (2006). Statins are associated with a reduced infrarenal abdominal aortic aneurysm growth. Eur J Vasc Endovasc Surg.

[21] Mosorin M, Juvonen J, Biancari F, Satta J, Surcel HM, Leinonen M (2001). Use of doxycycline to decrease the growth rate of abdominal aortic aneurysms: a randomized, double-blind, placebo-controlled pilot study. J Vasc Surg.

[22] Walton LJ, Franklin IJ, Bayston T, Brown LC, Greenhalgh RM, Taylor GW (1999). Inhibition of prostaglandin E2 synthesis in abdominal aortic aneurysms: implications for smooth muscle cell viability, inflammatory processes, and the expansion of abdominal aortic aneurysms. Circulation.

[23] Yoshimura K, Aoki H, Ikeda Y, Fujii K, Akiyama N, Furutani A (2005). Regression of abdominal aortic aneurysm by inhibition of c-Jun N-terminal kinase. Nat Med.

[24] Heart Protection Study Collaborative Group (2002). MRC/BHF heart protection study of cholesterol lowering with simvastatin in 20,536 high-risk individuals: a randomised placebo-controlled trial. Lancet.

[25] Ferguson CD, Clancy P, Bourke B, Walker PJ, Dear A, Buckenham T (2010). Association of statin prescription with small abdominal aortic aneurysm progression. Am Heart J.

[26] Daugherty A, Cassis L (2004). Angiotensin II and abdominal aortic aneurysms. Curr Hypertens Rep.

[27] Daugherty A, Manning MW, Cassis LA (2000). Angiotensin II promotes atherosclerotic lesions and aneurysms in apolipoprotein E-deficient mice. J Clin Invest.

[28] Rush C, Nyara M, Moxon JV, Trollope A, Cullen B, Golledge J (2009). Whole genome expression analysis within the angiotensin II-apolipoprotein E deficient mouse model of abdominal aortic aneurysm. BMC Genomics.

[29] Campos AH, Zhao Y, Pollman MJ, Gibbons GH (2003). DNA microarray profiling to identify angiotensin-responsive genes in vascular smooth muscle cells: potential mediators of vascular disease. Circ Res.

[30] Habashi JP, Doyle JJ, Holm TM, Aziz H, Schoenhoff F, Bedja D (2011). Angiotensin II type 2 receptor signaling attenuates aortic aneurysm in mice through ERK antagonism. Science.

[31] Golledge J, Muller J, Shephard N, Clancy P, Smallwood L, Moran C (2007). Association between osteopontin and human abdominal aortic aneurysm. Arterioscler Thromb Vasc Biol.

[32] Ahimastos AA, Aggarwal A, D‘Orsa KM, Formosa MF, White AJ, Savarirayan R (2007). Effect of perindopril on large artery stiffness and aortic root diameter in patients with Marfan syndrome: a randomized controlled trial. JAMA.

[33] Ahimastos AA, Dart AM, Kingwell BA (2008). Angiotensin II blockade in Marfan’s syndrome. N Eng J Med.

[34] Imayama I, Ichiki T, Inanaga K, Ohtsubo H, Fukuyama K, Ono H (2006). Telmisartan downregulates angiotensin II type 1 receptor through activation of peroxisome proliferator-activated receptor gamma. Cardiovasc Res.

[35] Sharma AM (2006). Telmisartan: the ACE of ARBs?. Hypertension.

[36] Jones A, Deb R, Torsney E, Howe F, Dunkley M, Gnaneswaran Y (2009). Rosiglitazone reduces the development and rupture of experimental aortic aneurysms. Circulation.

[37] Moran CS, Clancy P, Biros E, Blanco-Martin B, McCaskie P, Palmer LJ (2010). Association of PPARgamma allelic variation, osteoprotegerin and abdominal aortic aneurysm. Clin Endocrinol.

[38] Golledge J, Cullen B, Rush C, Moran CS, Secomb E, Wood F (2010). Peroxisome proliferator-activated receptor ligands reduce aortic dilatation in a mouse model of aortic aneurysm. Atherosclerosis.

[39] Moran CS, Cullen B, Campbell JH, Golledge J (2009). Interaction between angiotensin II, osteoprotegerin, and peroxisome proliferator-activated receptor-gamma in abdominal aortic aneurysm. J Vasc Res.

[40] Krishna SM, Seto SW, Moxon JV, Rush C, Walker PJ, Norman PE (2012). Fenofibrate increases high-density lipoprotein and sphingosine 1 phosphate concentrations limiting abdominal aortic aneurysm progression in a mouse model. Am J Pathol.

[41] Nishimoto M, Takai S, Fukumoto H, Tsunemi K, Yuda A, Sawada Y (2002). Increased local angiotensin II formation in aneurysmal aorta. Life Sci.

[42] Furubayashi K, Takai S, Jin D, Muramatsu M, Ibaraki T, Nishimoto M (2007). The significance of chymase in the progression of abdominal aortic aneurysms in dogs. Hypertens Res.

[43] Cruden NL, Witherow FN, Webb DJ, Fox KA, Newby DE (2004). Bradykinin contributes to the systemic hemodynamic effects of chronic angiotensin-converting enzyme inhibition in patients with heart failure. Arterioscler Thromb Vasc Biol.

[44] Messerli FH, Bangalore S, Ram VS (2008). Telmisartan, ramipril, or both in patients at high risk of vascular events. N Eng J Med.

[45] Iida Y, Xu B, Schultz GM, Chow V, White JJ, Sulaimon S (2012). Efficacy and mechanism of angiotensin II receptor blocker treatment in experimental abdominal aortic aneurysms. PLoS One.

[46] Matsumoto S, Kamide K, Banno F, Inoue N, Mochizuki N, Kawano Y (2010). Impact of RGS2 deficiency on the therapeutic effect of telmisartan in angiotensin II-induced aortic aneurysm. Hypertens Res.

[47] Kaschina E, Schrader F, Sommerfeld M, Kemnitz UR, Grzesiak A, Krikov M (2008). Telmisartan prevents aneurysm progression in the rat by inhibiting proteolysis, apoptosis and inflammation. J Hypertens.

[48] Thompson A, Cooper JA, Fabricius M, Humphries SE, Ashton HA, Hafez H (2010). An analysis of drug modulation of abdominal aortic aneurysm growth through 25 years of surveillance. J Vasc Surg.

[49] Parr A, Jayaratne C, Buttner P, Golledge J (2011). Comparison of volume and diameter measurement in assessing small abdominal aortic aneurysm expansion examined using computed tomographic angiography. Eur J Radiol.

[50] Moxon JV, Parr A, Emeto TI, Walker P, Norman PE, Golledge J (2010). Diagnosis and monitoring of abdominal aortic aneurysm: current status and future prospects. Curr Probl Cardiol.

[51] Golledge J, Muller R, Clancy P, McCann M, Norman PE (2011). Evaluation of the diagnostic and prognostic value of plasma D-dimer for abdominal aortic aneurysm. Eur Heart J.

[52] Sturm JW, Osborne RH, Dewey HM, Donnan GA, Macdonell RAL, Thrift AG (2002). Brief comprehensive quality of life assessment after stroke: the assessment of quality of life instrument in the north East melbourne stroke incidence study (NEMESIS). Stroke.

[53] Hawthorne G, Richardson J, Osborne R (1999). The assessment of quality of life (AQoL) instrument: a psychometric measure of health-related quality of life. Qual Life Res.

[54] Golledge J, Karan M, Moran CS, Muller J, Clancy P, Dear AE (2008). Reduced expansion rate of abdominal aortic aneurysms in patients with diabetes may be related to aberrant monocyte-matrix interactions. Eur Heart J.

[55] Telmisartan Randomised AssessmeNt Study in ACE iNtolerant subjects with cardiovascular Disease (TRANSCEND) Investigators, Yusuf S, Teo K, Anderson C, Pogue J, Dyal L, et al (2008). Effects of the angiotensin-receptor blocker telmisartan on cardiovascular events in high-risk patients intolerant to angiotensin-converting enzyme inhibitors: a randomised controlled trial. Lancet.

[56] Mann JFE, Schmieder RE, McQueen M, Dyal L, Schumacher H, Pogue J (2008). Renal outcomes with telmisartan, ramipril, or both, in people at high vascular risk (the ONTARGET study): a multicentre, randomised, double-blind, controlled trial. Lancet.

[57] DeMets DL, Lan KK (1994). Interim analysis: the alpha spending function approach. Stat Med.

[58] Imperial College London, Imperial Clinical Trials Unit. http://www1.imperial.ac.uk/clinicaltrialsunit/currenttrials/aardvark/ (accessed 24 December 2012).

[59] Hackam DG, Thiruchelvam D, Redelmeier DA (2006). Angiotensin-converting enzyme inhibitors and aortic rupture: a population-based case–control study. Lancet.

[60] Sweeting MJ, Thompson SG, Brown LC, Greenhalgh RM, Powell JT (2010). Use of angiotensin converting enzyme inhibitors is associated with increased growth rate of abdominal aortic aneurysms. J Vasc Surg.

