# Erratum to: ‘TElmisartan in the management of abDominal aortic aneurYsm (TEDY): The study protocol for a randomized controlled trial’

**DOI:** 10.1186/s13063-016-1183-x

**Published:** 2016-01-20

**Authors:** Dylan R. Morris, Margaret A. Cunningham, Anna A. Ahimastos, Bronwyn A. Kingwell, Elise Pappas, Michael Bourke, Christopher M. Reid, Theo Stijnen, Ronald L. Dalman, Oliver O. Aalami, Jan H. Lindeman, Paul E. Norman, Philip J. Walker, Robert Fitridge, Bernie Bourke, Anthony E. Dear, Jenna Pinchbeck, Rene Jaeggi, Jonathan Golledge

**Affiliations:** Queensland Research Centre for Peripheral Vascular Disease, School of Medicine and Dentistry, James Cook University, Townsville, QLD Australia; Psychology Department, University of Stirling, Stirling, UK; Baker IDI Heart and Diabetes Institute and The Department of Cardiovascular Medicine, Alfred Hospital Melbourne, Melbourne, Australia; Gosford Vascular Services, Gosford, New South Wales Australia; Centre of Cardiovascular Research and Education in Therapeutics, Department of Epidemiology and Preventive Medicine, Monash University, Alfred Hospital, Melbourne, Australia; Leiden University Medical Center, Leiden, The Netherlands; Division of Vascular Surgery, Department of Surgery, Stanford University School of Medicine, Stanford, CA USA; School of Surgery, University of Western Australia, Perth, WA Australia; University of Queensland School of Medicine, Discipline of Surgery and Centre for Clinical Research, and Department of Vascular Surgery, Royal Brisbane and Women’s Hospital, Brisbane, Queensland Australia; Department of Surgery, University of Adelaide, The Queen Elizabeth Hospital, Adelaide, South Australia Australia; Eastern Health Clinical School, Department of Medicine, Monash University, Melbourne, Australia; The Department of Vascular and Endovascular Surgery, The Townsville Hospital, Townsville, QLD Australia

Unfortunately, the original version of this article 1 [1] contained an error. Professor Jonathan Golledge is incorrectly referred to as deceased in the published article.

## Footnotes

1 Dylan R. Morris, Margaret A. Cunningham, Anna A. Ahimastos, Bronwyn A. Kingwell, Elise Pappas, Michael Bourke, Christopher M. Reid, Theo Stijnen, Ronald L. Dalman, Oliver O. Aalami, Jan H. Lindeman, Paul E. Norman, Philip J. Walker, Robert Fitridge, Bernie Bourke, Anthony E. Dear, Jenna Pinchbeck1, Rene Jaeggi and Jonathan Golledge: TElmisartan in the management of abDominal aortic aneurYsm (TEDY): The study protocol for a randomized controlled trial. Trials 2015, 16:274
